# Effects of different plantation years on grassland plant community in Maxian Mountain area of the Loess Plateau

**DOI:** 10.3389/fpls.2023.1123471

**Published:** 2023-02-14

**Authors:** Liang Mao, Jie Li, Xiao-Long Ma, Peter Quandahor, Yu-Ping Gou

**Affiliations:** ^1^College of Plant Protection, Gansu Agricultural University/Biocontrol Engineering Laboratory of Crop Diseases and Pests of Gansu Province, Lanzhou, China; ^2^Forestry and Grassland Bureau of Lintao County, Dingxi, Gansu, China; ^3^State Key Laboratory of Grassland Agro-ecosystems, College of Pastoral Agriculture Science and Technology, Lanzhou University, Lanzhou, China; ^4^Council of Scientific and Industrial Research (CSIR)- Savanna Agricultural Research Institute, Tamale, Ghana

**Keywords:** The Loess Plateau, artificial afforestation, plantation years, grassland plant community, ecosystems restoration

## Abstract

Plant communities in the Loess Plateau’s artificial afforestation forests play an important role in fragile ecosystem restoration. Therefore, the composition, coverage, biomass, diversity, and similarity of grassland plant communities in different years of artificial afforestation in cultivated land were investigated. The effects of years of artificial afforestation on grassland plant community succession in the Loess Plateau were also investigated. The results showed that as the number of years of artificial afforestation increased, grassland plant communities grew from scratch, constantly optimizing community components, improving community coverage, and increasing aboveground biomass. The community diversity index and similarity coefficient gradually approached those of a 10-year abandoned community that had recovered naturally. After 6 years of artificial afforestation, the dominant species of the grassland plant community changed from *Agropyron cristatum* to *Kobresia myosuroides*, and the main associated species changed from Compositae and Gramineae to Compositae, Gramineae, Rosaceae, and Leguminosae. The α-diversity index accelerated restoration, the richness index and diversity index increased, and the dominant index decreased. The evenness index had no significant difference from CK. The β-diversity index decreased as the number of years of afforestation increased. The similarity coefficient between CK and grassland plant communities in various lands changed from medium dissimilarity to medium similarity at 6 years of afforestation. According to the analysis of various indicators of the grassland plant community, the grassland plant community had a positive succession within 10 years of artificial afforestation on the cultivated land of the Loess Plateau, and the threshold of the years from slow to fast was 6 years.

## Introduction

The Loess Plateau is the world’s largest loess accumulation area, with an area of 620,000 km^2^ and a soil layer thickness of 80–120 m (Huang et al., 2020; Tang et al., 2010). It has been intensively eroded by running water for a long time, with thousands of ravines, continuous loss of surface mature soil, bare raw soil, and poor fertility, as one of the regions with the most serious water and soil loss in the world (Schnitzer et al., 2013; Zhu et al., 2018). The Loess Plateau, located in the Yellow River’s middle and upper reaches, is an important birthplace of Chinese civilization. It has a long farming history but is sparsely forested and grass-covered, with significant soil and water loss (Xia et al., 2021). It is the Yellow River’s primary water supply and sediment input area (Zhang et al., 2017). Therefore, the fragile ecosystem of the Loess Plateau has been reduced from the origin of agricultural civilization to a poor area of economic development, severely limiting Chinese society’s sustainable development (Li et al., 2016). In 1999, Loess Plateau in Gansu, Shanxi, and Sichuan became a key artificial afforestation area for the project after China took the lead in implementing the pilot project of returning farmland to forests (Liu et al., 2017; Li and Liu, 2022). Over the last 20 years, China has converted 33.9 million km^2^ of land to forests and grasslands, and the vegetation coverage of the Loess Plateau has increased from 27.4% in 2000 to 57.5% in 2019 (Song et al., 2022).

The purpose of reforesting farmland on the Loess Plateau is to increase vegetation coverage, reduce water and soil loss, and restore the ecological environment by reforesting farmland with significant water and soil loss, difficult cultivation, and low grain production (Guo et al., 2018; Wein, 1986). However, the Loess Plateau’s natural conditions, which include low temperature, little rainfall, thick soil layer, and, in particular, drought and water scarcity, severely limit vegetation growth (Wang et al., 2022). Therefore, in order to achieve a one-sided survival rate of artificial afforestation on the Loess Plateau (Deng et al., 2017), the tree species such as spruce, locust, and apricot are typically used (Dong and Li 2013). Large-scale and extensive ecological environment restoration could be accomplished by using the time-saving and labor-saving pure forest afforestation model (Bennett et al., 2014), the dogmatic planting of a single tree species into the cultivated land, and then taking measures to close the mountain for afforestation (Wang et al., 2001). This resulted in a mismatch between the artificial afforestation tree species and the natural conditions on the Loess Plateau, which accelerated water evaporation in the artificial afforestation area (Wang et al., 2011), promoted water scarcity in local areas, and caused the phenomenon of dry stratification of soil (Chen et al., 2007), resulting in the problem of small and old trees with weak growth, low growth, and weak growth of a single artificially planted tree species (Han and Hou, 1996). With the number of years of artificial afforestation, the Loess Plateau is increasingly covered with artificial forest land with unreasonable water allocation, low ecological restoration function, and low canopy density (Yu and Jiao, 2018).

The herbaceous vegetation beneath the arbor canopy is an important component of artificial afforestation and plays a unique role in regulating soil moisture, maintaining biodiversity, and stabilizing the ecosystem (Marcelo et al., 2014). *Picea* is a widely representative planting species for artificial afforestation (Yue et al., 2018). For a long time, the spruce planted in the Loess Plateau cultivated land has been subjected to drought stress, low temperature, barren, and other stresses. It matures slowly into a small old tree. However, as the number of years of artificial afforestation increased, so did the grassland plant community under the spruce canopy (Cui et al., 2014; Zhang et al., 2017). Therefore, studying the succession law of grassland plant communities under the spruce canopy in the artificial forestation area of cultivated land in the Loess Plateau to consolidate and enhance the project of returning farmland to forest and grassland is paramount. This study is based on the hypothesis that different years of afforestation will affect grassland plant communities. This study was therefore conducted to determine the effects of years of artificial afforestation on the succession of grassland plant communities in the Loess Plateau.

## Materials and methods

### Plant material and growing conditions

The study was conducted in Maxian Mountain on the Loess Plateau (geographic coordinates 35°45′N, 103°58′E, altitude 2584-2864 m, average temperature is -1.4°C, frost-free period 67–90 days, average annual rainfall 494 mm), which is approximately 40 km south of Lanzhou urban area (Mu et al., 2017). The climate is an alpine meadow climate (Dong et al., 2013). The land is subalpine shrub soil dominated by *Rhododendron qinghaiense* and *Hippophae rhamnoides* subspecies, as well as associated species, such as *Lonicera hispida* and *Arctous rubra* (Sun and Zhao, 1995).

### Sample plot setting and investigation

**Figure 1:**
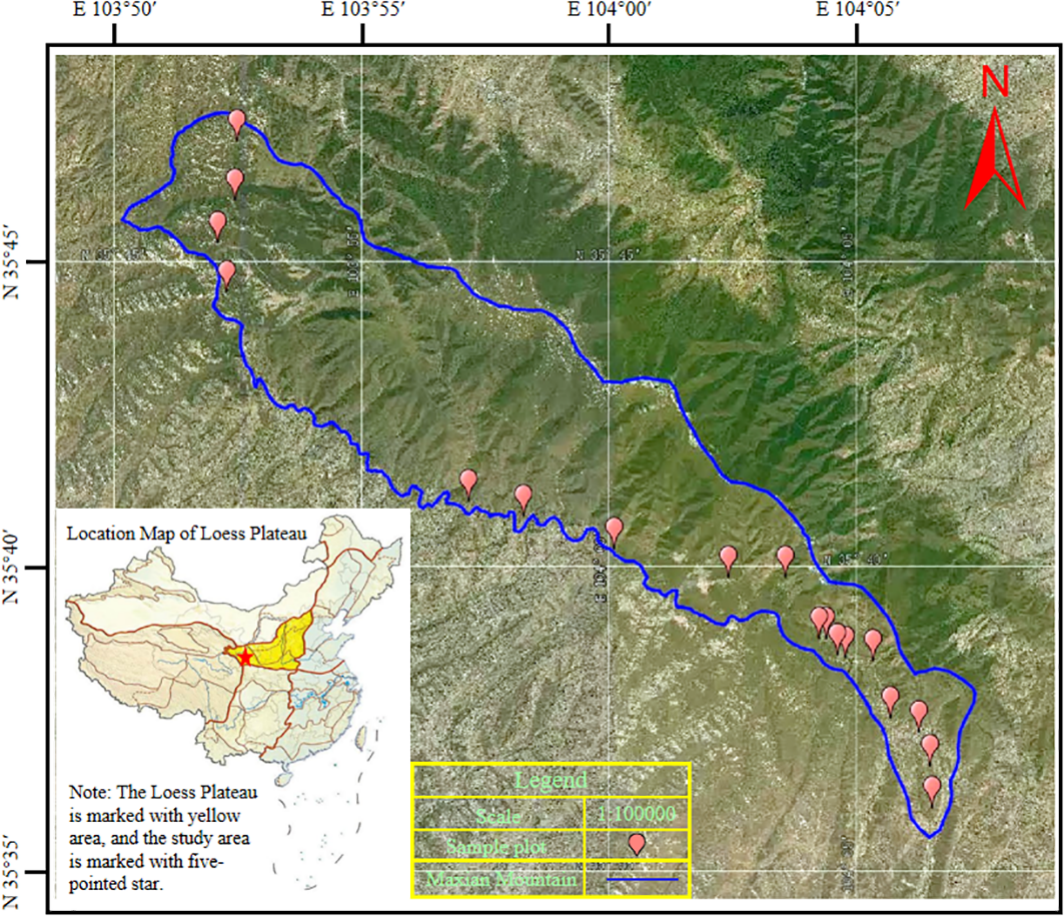
Map of research area and sample plot distribution.

The study area is located in Lintao County, Dingxi City, Gansu Province, from Houdiwan Village, Taishi Town, west of Maxian Mountain, to Puyin Road, Pujisi Village, Xiakou Town, east of Maxian Mountain. The tree species planted in the cultivated land was spruce with a row spacing of 2 m× 3 m according to the project of returning farmland to forests implemented in 2010, 2012, 2014, 2016, 2018, and 2020. The research object was a wire-fenced artificial forestation land. Gradients of 10, 8, 6, 4, and 2 years of artificial afforestation were formed in turn, and then CK was then planted in sample plots where the cultivated land had been abandoned for 10 years and had recovered naturally, for a total of six treatments. There were three 10 m × 10 m sample plots for each treatment, for a total of 18 plots. See **Figure 1** for the map of the research area and sample plot distribution.

There were no shrubs on the artificial forestation land. *Picea asperata* Mast had an average height of 1.5–2.0 m, a canopy density of less than 0.2, and slope aspect and elevation that were basically identical (**Table 1**). In each sample plot, the quadrat was arranged in three 1 m× 1 m rows along the “S” shape. Each quadrat was located in the center of four *P. asperata* Mast, and the species composition, density, coverage, frequency, and other characteristics of grassland plant communities in the quadrat were counted before the aboveground biomass was harvested and taken to the laboratory for drying and weighing. A total of 54 samples were examined, and the field survey was completed between July and August 2022.

**Table 1:** Basic information of sample plots with different years of artificial afforestation in the Maxian Mountains of the Loess Plateau.

Plot no.	Artificial afforestation (year)	Geographic coordinates	Aspect	Altitude (m)
1	CK	35°38′48″N 104°04′5″E	North	2,790
2	CK	35°38′26″N 104°05′11″E	Northwest	2,719
3	CK	35°46′01″N 103°52′19″E	North	2,632
4	10	35°40′17″N 103°59′58″E	North	2,775
5	10	35°37′16″N 104°06′06″E	Northwest	2,628
6	10	35°38′48″N 104°04′14″E	Northwest	2,776
7	8	35°44′31″N 103°52′09″E	Northeast	2,738
8	8	35°36′43″N 104°06′20″E	North	2,584
9	8	35°38′32″N 104°04′28″E	Northeast	2,766
10	6	35°36′02″N 104°06′22″E	Northwest	2,606
11	6	35°39′49″N 104°03′25″E	Northwest	2,846
12	6	35°45′19″N 103°51′58″E	North	2,704
13	4	35°46′59″N 103°52′21″E	Northwest	2,804
14	4	35°38′29″N 104°04′37″E	North	2,765
15	4	35°40′50″N 103°58′08″E	Northeast	2,710
16	2	35°37′30″N 104°05′32″E	North	2,595
17	2	35°39′49″N 104°02′16″E	Northeast	2,783
18	2	35°41′05″N 103°57′01″E	Northwest	2,694

### Index calculation

The α diversity was selected for the Patrick richness index (S), Shannon–Wiener diversity index (H), Simpson dominance index (D), and Pielou evenness index (J). These were calculated as S = total number of species in the quadrat; *H*=−∑*P*_*i*_*lnP*_*i*_ ; $D=\sum P_{1}^{2}$ ; *J*=*H*/*lnS* , where *P*_i_ is the important value of species i, *P*_i_ = (relative coverage + relative density + relative frequency)/3 (Mao et al., 2013).

The β diversity was selected for the Whittaker index (β w). It was calculated as *β*_*w*_=(*S*/*X*)−1 , where *S* is the cumulative number of species in all sample plots and *X* is the species richness in the quadrat (Li et al., 2021).

Community similarity (*C_J_*) uses the Jaccard similarity coefficient, and it was calculated as $C_{J}=\frac{c}{a+\text{b}-\text{c}}$ , where *a* and *b* represent the total number of species in communities A and B, respectively, and *c* represents the number of species common to the two communities (He et al., 2017).

### Data processing

ANOVA and Excel drawing were conducted with SPSS 17.0 software.

## Results

### Effects of different afforestation years on the composition of grassland plant communities

The grassland plant community’s dominant species and associated species in the Loess Plateau artificial afforestation area changed as the years of artificial afforestation increased (**Table 2**). In terms of grassland plant community dominant species, *Agropyron cristatum* was planted for 2 and 4 years, and *Carex myosuroides* was planted for 6, 8, and 10 years, which was the same as the CK. In terms of associated species of the grassland plant community, there were 18 species in 2 years belonging to 15 genera and 9 families, 21 species in 4 years belonging to 19 genera and 9 families, 32 species in 6 years belonging to 26 genera and 13 families, 34 species in 8 years belonging to 29 genera and 14 families, 41 species in 10 years belonging to 35 genera and 15 families, and 46 species in CK belonging to 38 genera and 17 families. Asteraceae, Poaceae, Rosaceae, Fabaceae, and Gentianaceae dominated artificial afforestation CK and associated species for 10 years. Asteraceae, Poaceae, Rosaceae, and Fabaceae dominated in 8 and 6 years. Asteraceae and Poaceae dominated in 4 and 2 years. Therefore, the dominant species of grassland plant community has changed from *A. cristatum* to *C. myosuroides*, and the main associated species have also changed from Asteraceae and Poaceae to Asteraceae, Poaceae, Rosaceae, Fabaceae, and Gentianaceae. The number of species in the grassland plant community has increased.

**Table 2:** Grassland plant community components of different artificial afforestation years in the Maxian Mountain area of the Loess Plateau.

Artificial afforestation (year)	Dominant species	Companion species
CK	*Carex myosuroides*	*Agropyron cristatum*, *Stipa breviflora*, *P. pratensis*, *E. nutans*, *Geranium wilfordii*, *Festuca elata*, *Potentilla supina*, *Knorringia sibirica*, *Ligularia sagitta*, *Medicago ruthenica*, *P. sericea*, *Saussurea pulchra*, *O. kansuensis*, *Taraxacum mongolicum*, *G. straminea*, *Allium mongolicum*, *A. capillaris*, *Nepeta cataria*, *Ixeris polycephala*, *Ajania tenuifolia*, *Elsholtzia densa*, *Dysphania schraderiana*, *Iris lactea*, *Aster tataricus*, *Euphorbia esula*, *Clematis florida*, *Anaphalis lactea*, *Gymnaconitum gymnandrum*, *Leontopodium nanum*, *Pedicularis sylvatica*, *Gentiana dahurica*, *Thermopsis lupinoides*, *Aster altaicus*, *Gentiana scabra*, *Gentianopsis barbata*, *Artemisia stechmanniana*, *Artemisia argyi*, *Lancea tibetica*, *Artemisia hedinii*, *Sanguisorba officinalis*, *Plantago asiatica*, *Cirsium spicatum*, *Cirsium arvense*, *Neotrinia splendens*, *Carduus nutans*, *Androsace umbellata*, etc. 46 species
10	*C. myosuroides*	*A. cristatum*, *P. pratensis*, *F. elata*, *P. supina*, *S. breviflora*, *E. nutans*, *G. wilfordii*, *O. kansuensis*, *P. sericea*, *K. sibirica*, *M. ruthenica*, *S. pulchra*, *I. polycephala*, *A. mongolicum*, *A. capillaris*, *D. schraderiana*, *N. cataria*, *Scutellaria baicalensis*, *E. densa*, *A. tenuifolia*, *A. tataricus*, *N. splendens*, *L. sagitta*, *A. altaicus*, *A. stechmanniana*, *C. arvense*, *A. umbellata*, *L. nanum*, *P. sylvatica*, *G. dahurica*, *A. argyi*, *Calystegia hederacea*, *T. lupinoides*, *P. asiatica*, *A. hedinii*, *Tibetia himalaica*, *G. barbata*, *S. officinalis*, *Cicuta virosa*, *C. spicatum*, *C. nutans*, etc. 41 species
8	*C. myosuroides*	*A. cristatum*, *S. breviflora*, *A. altaicus*, *P. supina*, *G. wilfordii*, *O. kansuensis*, *A. mongolicum*, *T. mongolicum*, *K. sibirica*, *M. ruthenica*, *A. capillaris*, *E. nutans*, *A. tenuifolia*, *L. nanum*, *P. pratensis*, *G. gymnandrum*, *S. pulchra*, *P. sericea*, *A. argyi*, *A. stechmanniana*, *A. hedinii*, *G. straminea*, *C. florida*, *N. cataria*, *C. arvense*, *T. lupinoides*, *L. tibetica*, *P. asiatica*, *S. officinalis*, *N. splendens*, *Kali collinum*, *C. hederacea*, *C. spicatum*, *G. barbata*, etc. 34 species
6	*C. myosuroides*	*A. cristatum*, *S. breviflora*, *K. sibirica*, *M. ruthenica*, *A. capillaris*, *T. mongolicum*, *G. wilfordii*, *A. tenuifolia*, *A. altaicus*, *P. supina*, *S. pulchra*, *L. nanum*, *E. nutans*, *A. argyi*, *A. mongolicum*, *A. hedinii*, *P. pratensis*, *Stellera chamaejasme*, *P. sericea*, *A. lactea*, *O. kansuensis*, *Daphne tangutica*, *A. stechmanniana*, *C. arvense*, *A. umbellata*, *A. tataricus*, *P. asiatica*, *C. hederacea*, *P. sylvatica*, *S. officinalis*, *G. scabra*, *C. spicatum*, etc. 32 species
4	*A. cristatum*	*F. elata*, *K. sibirica*, *I. polycephala*, *A. lactea*, *S. chamaejasme*, *A. altaicus*, *A. hedinii*, *A. capillaris*, *P. pratensis*, *A. stechmanniana*, *I. lactea*, *T. mongolicum*, *O. kansuensis*, *M. ruthenica*, *E. nutans*, *N. cataria*, *A. tenuifolia*, *N. splendens*, *P. asiatica*, *C. arvense*, *P. sylvatica*, etc. 21 species
2	*A. cristatum*	*S. chamaejasme*, *S. pulchra*, *T. mongolicum*, *E. densa*, *A. hedinii*, *A. altaicus*, *A. stechmanniana*, *A. capillaris*, *A. tataricus*, *C. florida*, *A. tenuifolia*, *M. ruthenica*, *K. collinum*, *C. hederacea*, *N. splendens*, *C. arvense*, *P. sylvatica*, *I. polycephala*, etc. 18 species

The important values of associated species in the table decrease in turn.

### Effects of different afforestation years on the coverage of grassland plant communities

**Figure 2:**
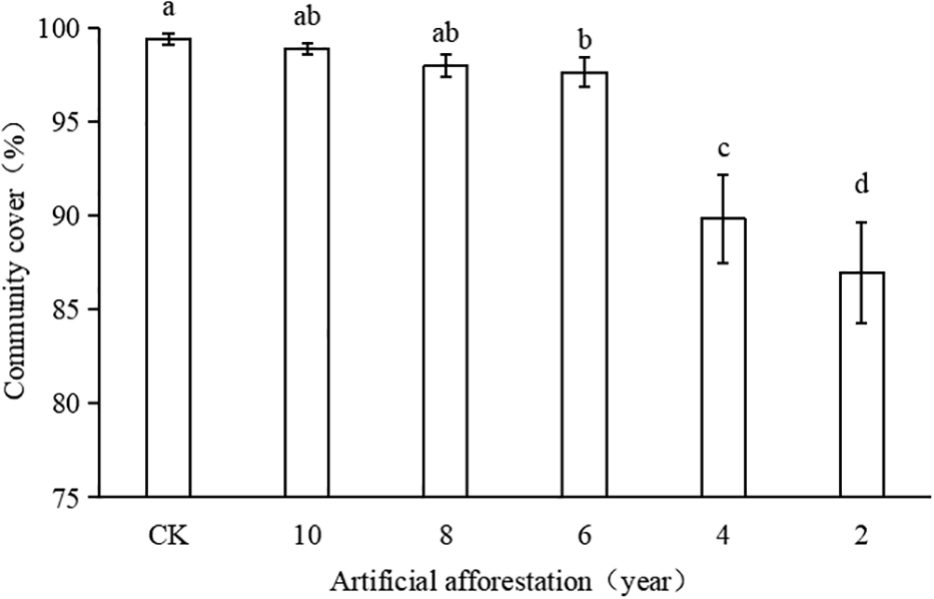
Coverage of grassland plant communities with different artificial afforestation years in Maxian Mountain of the Loess Plateau. Different lowercase letters indicate statistically significant differences in coverage of grassland plant communities between artificial afforestation years by LSD test (p< 0.05).

In the Loess Plateau’s artificial forestation area, the coverage of the grassland plant community gradually increased with increasing years of artificial forestation (**Figure 2**). The years of artificial afforestation were 2, 4, 6, 8, and 10 years, with the coverage of grassland plant community being 86.9%, 89.8%, 97.7%, 98.0%, and 98.9%, respectively, and that of the CK grassland plant community was 99.4%. When the artificial afforestation years were 10, 8, and 6 years, grassland plant community coverage was significantly higher than when the artificial afforestation years were 4 and 2 years (p< 0.05), whereas when the artificial afforestation years were 10 and 8 years, there was no significant difference between grassland plant community cover and CK. It demonstrates that the artificial afforestation period was 6 years, and the grassland plant community coverage was close to CK.

### Effects of different afforestation years on aboveground biomass of grassland plants

**Figure 3:**
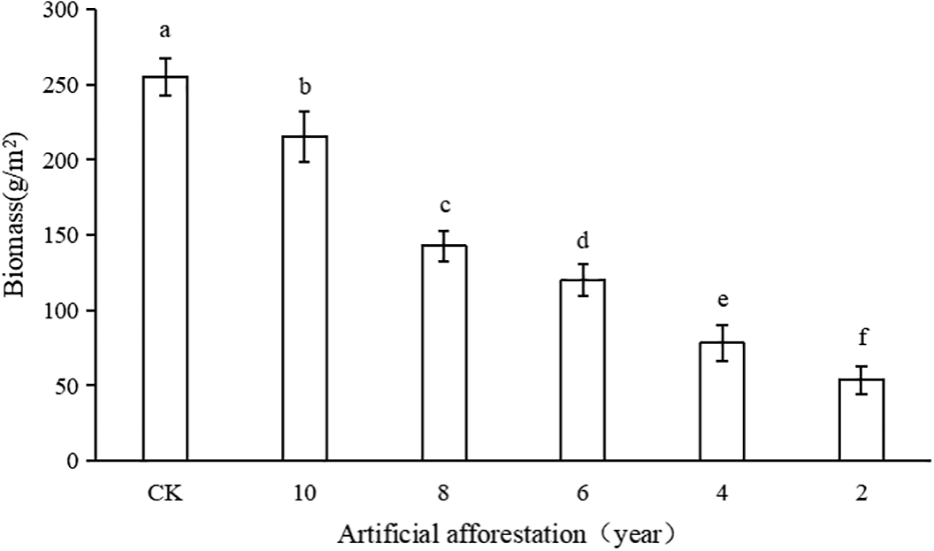
Aboveground biomass of grassland plants with different years of artificial afforestation in the Maxian Mountains of the Loess Plateau. Different lowercase letters indicate statistically significant differences in aboveground biomass of grassland plants between artificial afforestation years by LSD test (p< 0.05).

In the Loess Plateau artificial afforestation area, the aboveground biomass of grassland plants increased as the number of years of artificial afforestation increased (**Figure 3**). The aboveground biomass of grassland plants in the sample plots with artificial afforestation years of 2, 4, 6, 8, and 10 years was 53.54, 78.09, 119.81, 142.61, and 215.28 g/m^2^, respectively, which were significantly lower than in CK sample plots (p< 0.05). However, when the afforestation period was 10 years, CK had 84% aboveground biomass of grassland plants, whereas when the afforestation period was 8, 6, 4, and 2 years, the grassland plants’ aboveground biomass was 56%, 47%, 31%, and 21% of CK, respectively. It shows that the grassland plants’ aboveground biomass increased slowly in the early stages of artificial afforestation and rapidly in the later stages of 10 years, but it was significantly smaller than CK.

### Effects of different plantation years on the diversity of grassland plant communities

#### α Diversity

In the Loess Plateau artificial afforestation area, the grassland plant community α-diversity index gradually approached CK as the artificial afforestation number of years increased (**Table 3**). As the artificial afforestation years increased, the richness and diversity indexes of the grassland plant community gradually increased but significantly lower than those of CK, and the dominance index gradually decreased but was significantly (p< 0.05) higher than CK. The diversity and dominance indexes of grassland plant communities are significantly different from each other except when the afforestation years were 6 and 8 years. With increasing years of artificial afforestation, the evenness index of the grassland plant community increased, then decreased, and then increased. The sample plots with 6 years of artificial afforestation and 2 years of artificial afforestation had the maximum and minimum values, respectively. There was no significant difference between CK and the sample plots with 10 and 6 years of artificial afforestation, but they were significantly greater than the sample plots with 8, 4, and 2 years of artificial afforestation. It shows that the richness, diversity, dominance, and evenness indexes of the grassland plant community gradually recovered after 10 years of artificial afforestation, but when the artificial afforestation period was 6 years, the α diversity accelerated its recovery in the grassland plant community.

**Table 3:** α-Diversity index of grassland plant communities with different artificial afforestation years in Maxian Mountain of the Loess Plateau.

Artificial afforestation (year)	Richness index (S)	Diversity index (H)	Dominant index (D)	Evenness index (J)
CK	44 ± 1.2247a	3.5701 ± 0.0367a	0.0335 ± 0.0023e	0.9437 ± 0.0077ao
10	38 ± 1.8708b	3.3937 ± 0.0277b	0.0405 ± 0.0019d	0.9334 ± 0.0135ab
8	30 ± 2.3979c	3.1618 ± 0.0631c	0.0492 ± 0.0043c	0.9305 ± 0.0122bc
6	28 ± 2.2361d	3.1102 ± 0.0565c	0.0507 ± 0.0033c	0.9343 ± 0.0103ab
4	19 ± 1.8708e	2.7101 ± 0.0863d	0.0684 ± 0.0055b	0.9219 ± 0.0094co
2	15 ± 1.5000f	2.4581 ± 0.0779e	0.0828 ± 0.0052a	0.9095 ± 0.0136d

Different lowercase letters indicate statistically significant differences in α-diversity index of grassland plant between artificial afforestation years by LSD test (p< 0.05).

#### β Diversity

**Figure 4:**
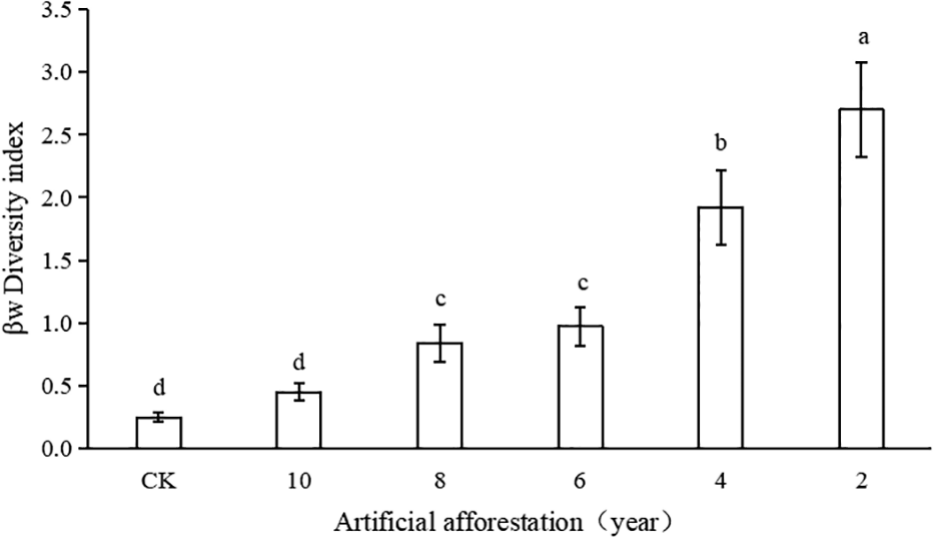
β-Diversity index of grassland plant communities with different years of artificial afforestation in the Maxian Mountains of the Loess Plateau. Different lowercase letters indicate statistically significant differences in β-diversity index of grassland plant communities between artificial afforestation years by LSD test (p< 0.05).

In the Loess Plateau artificial afforestation area, the grassland plant community’s β-diversity index gradually decreased and approached CK as the number of years of artificial afforestation increased (**Figure 4**). Grassland plant communities in sample plots with 2 and 4 years’ β artificial afforestation years had a significantly higher diversity index than the sample plots with 6 and 8 years’ afforestation. The β-diversity index showed no significant difference between the grassland plant community and CK after 10 years of afforestation (p< 0.05). It shows that as the number of years of artificial afforestation increased, the grassland plant community of the β-diversity index recovered gradually. When the afforestation period was 4–6 years, the β-diversity index rose rapidly.

### Effects of different plantation years on the similarity of grassland plant communities

In the Loess Plateau artificial afforestation area, grassland plant communities with different artificial afforestation years were similar to each other to varying degrees (**Table 4**). The Jaccard similarity principle showed community similarity coefficient intervals of [0.00, 0.25], [0.25, 0.50], [0.50, 0.75], and [0.75, 1.00], representing very dissimilar, medium dissimilar, medium similar, and very similar, respectively (He et al., 2017). The artificial afforestation years were 2 and 4 years, with a medium relationship between the grassland plant community and the various plots. There was a medium similarity between CK and grassland plant communities in the sample plots with 6, 8, and 10 years of artificial afforestation. It is an indication that when the artificial afforestation period is 6 years or more, the similarity coefficient of the grassland plant community changed from a medium non-similarity relationship to a medium similarity relationship, and the similarity of the grassland plant community increased.

**Table 4:** Similarity coefficient of grassland plant community with different years of artificial afforestation in Maxian Mountain of the Loess Plateau.

Artificial afforestation (year)	CK	10	8	6	4	2
CK	1.0000					
10	0.7451	1.0000				
8	0.6735	0.6383	1.0000			
6	0.6000	0.5957	0.6190	1.0000		
4	0.4375	0.3913	0.3902	0.4474	1.0000	
2	0.3200	0.3261	0.3500	0.3684	0.4643	1.0000

## Discussion

Afforestation is one of the main strategies for improving the fragile ecosystem, and the under-storied grassland plant community is playing an important role in stabilizing the ecosystem through positive succession as the number of years increases (Li et al., 2023). The present study showed that after artificial afforestation in the farmland of Maxian Mountain on the Loess Plateau, the dominant species of grassland plant community optimized from *A. cristatum* to *C. myosuroides*, and the main associated species gradually increased from Asteraceae and Poaceae to Asteraceae, Poaceae, Rosaceae, Fabaceae, and Gentianaceae. The number of plant species ranged from few to many, the community components were complex and diverse, and community coverage ranged from low to high. The composition and coverage of grassland plant communities gradually resembled those that had recovered naturally after 10 years of abandonment of cultivated land when the artificial afforestation lasted 6 years or more, which is consistent with the vegetation succession trend of Liu Shuo’s conversion of farmland to forest land in the Loess Plateau’s hilly and gully regions (Liu and He 2006). Apparently, after 5 years of artificial afforestation, the grassland plant communities appear to have transitioned from a slow positive to a fast positive succession stage. This is possible because in the early stages of artificial afforestation in cultivated land, the exposed topsoil, while loose in texture, has a high amount of soil evaporation due to wind and sun exposure, especially the low water content in the shallow layer of the soil, and the obvious drying of the soil. Moreover, the soil seed bank is depleted, and the local grass species can only enter the soil for germination and growth through natural transmission channels such as wind and water flow. At this time, the habitat is not suitable for new plants to obtain soil nutrients and water. In harsh environments, they die quickly, and only plants with high resistance can survive. Therefore, the drought-tolerant, cold-tolerant, and barren-tolerant *A. cristatum* geminated and reproduced earlier to become the dominant species, and the associated species only survived the stress-resistant plants such as the *Saussurea pulchra* of Asteraceae and *Neotrinia splendens* of Poaceae. The number of years of artificial afforestation increased the grassland plants’ coverage on previously bare farmland, improved the surface, made the habitat milder, and improved the ability to store water and retain moisture. *C. myosuroides*, which prefers shade and humidity, gradually displaced *A. cristatum* as the dominant species, and additional species also emerged. Rosaceae, Fabaceae, and Gentianaceae, among others, gradually succeeded as companion species. The composition of the grassland plant community was diversified, and the coverage was stable, similar to the natural recovery community.

The aboveground biomass of the grassland plant community directly reflects the size of productivity (Wang et al., 2009). In the present study, the aboveground biomass of grassland increased as the number of years of artificial afforestation increased, and the grassland productivity gradually recovered, but the degree of recovery varied over time, exhibiting slow to fast characteristics. This is consistent with the findings of Shi Yangyang and other findings in Ansai County, Shanxi Province, who discovered that abandoned farmland continued to recover grassland plant productivity over time (Shi et al., 2012). This is because once human disturbance is removed from cultivated land, the grassland plant community begins to recover naturally, and aboveground biomass increases (Su et al., 2021). However, the grassland plant community has a flexible biomass allocation strategy in which biomass is reasonably allocated based on the efficiency of light, temperature, water, nutrients, and other resources, which is adaptable to environmental changes and meets the growth requirements (Hui and Jackson, 2006). The slow growth of grassland aboveground biomass in the early stages of artificial afforestation in cultivated land is due to the bare ground surface and other rich resources, whereas underground water, nutrients, and other resources become limiting factors. Herbaceous plants may allocate more biomass to their root systems in order to obtain more underground resources, ensuring their survival. The growth of grassland aboveground biomass was clearly accelerated when the artificial afforestation period was 6 years or longer and gradually approached the natural recovery community. This is due to the grassland community’s many years of positive succession, and the community structure is complex and diverse. Not only did aboveground biomass have a significant superposition accumulation effect when compared with the previous period, but ground light and temperature resources also became the main limiting factors. Herbs allocate more biomass to branches, leaves, and other aboveground parts during the growth process (Franco et al., 2020). Under the same conditions, the increase in aboveground biomass is usually greater than the underground biomass, causing the acceleration of grassland productivity.

Plant diversity is essential for grassland ecosystem stability (Li et al., 2022). In the present study, as the number of years of artificial afforestation increased, the α-diversity index of the grassland plant community in the artificial afforestation area of the farmland in the Maxian Mountain on the Loess Plateau gradually approached CK. The richness and diversity indexes increased, the dominance index decreased, and the evenness index first increased, then decreased, and finally increased. This is due to the intention of artificial afforestation in cultivated land to transform the originally regulated farmland ecosystem into the naturally restored forest grass ecosystem. This sudden change is bound to release more niches, improving the living conditions of herbaceous plants. After 2–4 years of artificial afforestation in the cultivated land, the surface soil is loose, some opportunistic species are rapidly settled and propagated, and the pioneer grass species occupy a relatively wide niche (Mao et al., 2013), forming a grassland plant community with simple structure and prominent dominant species, resulting in high dominance index and low richness and diversity indexes, building an unbalanced community structure (Li et al., 2021). At this time, the community evenness index is low. However, when the artificial afforestation period is 6 years or more, the increase in coverage, density, and aboveground biomass of grassland plant communities can intercept precipitation and weaken the erosion effect of precipitation, thus promoting the function of soil conservation and water conservation, and the accumulation of soil nutrients is also increasing (Cui et al., 2014). Meanwhile, the environmental heterogeneity is reduced, which meets the niche needs of more species, and the probability of occurrence of each species is small (Zhang et al., 2017). In addition, the composition of the community structure was optimized and adjusted to form a stable grassland plant community with a low dominance index and increased richness and diversity indexes. The evenness index of the grassland plant community is the largest when the artificial afforestation period is 6 years. This is because at this time, the dominant species is changed from ice grass to kobresia, the intra-specific competition is reduced, and only some associated species compete with each other for niche space, causing the evenness index to fluctuate slightly, but there is no significant difference.

The β diversity reflects the process of changing community species composition along a certain environmental gradient, revealing the rate of species substitution and species nesting among communities (Li et al., 2021). In the present study, the grassland plant community in the cultivated land in the Maxian Mountain on the Loess Plateau increases with the number of years of artificial afforestation, while the β-diversity index steadily decreased. When the afforestation period is 6 years, the decline rate gradually approaches CK from fast to slow, indicating that the replacement rate of grassland vegetation community decreases and there are species nesting. This is due to the obvious environmental heterogeneity of different afforestation years, which affects the composition of the grassland plant community. In the early stages of artificial forestation, the undergrowth vegetation is scarce, and pioneer grass species have priority effects, occupying more ecological niches and repelling the subsequent species. However, the subsequent grass species continue to successfully settle and compete for limited niche resources, the interspecific competition is fierce, and the species replacement rate of the grassland vegetation community is rapid. After 6 years of artificial afforestation, the grassland plant community species composition becomes rich and diverse, habitat heterogeneity decreases, and species with a high tolerance for environmental change can be retained. Grassland communities with fewer species become subsets of grassland communities with more species, demonstrating a nested pattern.

The similarity coefficient of plant communities is an important indicator to reflect community heterogeneity as well as a specific manifestation of community habitat heterogeneity (He et al., 2017). The present results showed that after 6 years of afforestation, the community similarity index of the grassland plant community and CK community changed from medium dissimilarity to medium similarity. This indicates that because the artificial afforestation period was 6 years or longer, the heterogeneity of habitat conditions was weakened, and the undergrowth grassland plant community recovered naturally. The dominant species were the same as in CK, and the associated species were similar to CK, but with a greater number of species. This also confirms the grassland plant community. The diversity index decreased gradually and steadily along the environmental gradient of increasing afforestation years.

## Conclusion

The results of this study showed that within the 10-year time span, the artificial afforestation year was 6 years, which is the time threshold of the grassland plant community from quantitative to qualitative change. When compared to the sample plots with only 4 and 2 years of artificial afforestation, the composition, coverage, and aboveground biomass of the grassland plant community were significantly restored. The α-diversity index accelerated its recovery, and the β-diversity index decreased and changed from species substitution to species nesting, affecting the similarity coefficient of the grassland plant community and the community that had undergone recovery naturally. However, the 10-year artificial afforestation year’s gradient in this study is insufficient to fully reveal the succession law of grassland plant communities due to its short duration. It would be necessary to further investigate the impact of more than 10 years of artificial afforestation on grassland plant communities through controlled experiments, as this will provide scientific evidence for the restoration of grassland plant communities.

## Data availability statement

The original contributions presented in the study are included in the article/**Supplementary Material**. Further inquiries can be directed to the corresponding author.

## Author contributions

LM and Y-PG contributed to study concept, design and statistical analysis. LM, JL, X-LM, and Y-PG contributed to investigation and resources. LM and Y-PG contributed to drafting of the manuscript. PQ contributed to review, editing and proofreading of the manuscript. Y-PG contributed to funding acquisition and study supervision. All authors contributed to the article and approved the submitted version.
